# Procedural or declarative deficit in adults with developmental dyslexia? A study of Artificial grammar learning

**DOI:** 10.1371/journal.pone.0352337

**Published:** 2026-07-29

**Authors:** Giuliana Nardacchione, Pierluigi Zoccolotti, Chiara Valeria Marinelli

**Affiliations:** 1 Cognitive and Affective Neuroscience Laboratory, Department of Humanities, Letters, Cultural Heritage and Educational Studies, Foggia University, Foggia, Italy; 2 Department of Psychology, Sapienza University of Rome, Rome, Italy; 3 Tuscany Rehabilitation Clinic, Montevarchi, Italy; 4 Gianfranco Salvini Foundation for Rehabilitation Research, Montevarchi, Italy; Basque Center on Cognition Brain and Language, SPAIN

## Abstract

Artificial grammar learning (AGL) has frequently been employed to investigate the procedural hypothesis of dyslexia. However, most studies did not distinguish whether performance depended upon the acquisition of grammatical rules (procedural memory), distributional knowledge (statistical learning) or reference to individual memory traces (instances). An AGL paradigm was administered to 32 young adults with dyslexia and 60 controls. During the learning phase, adults with dyslexia learned artificial grammar and then utilized procedural memory in the grammaticality judgment and recognition tasks to a similar extent as controls. An overall group difference was observed only on the grammaticality judgment task; however, the performance of both groups was similarly modulated by the grammaticality of the stimuli. Adults with dyslexia acquired and used individual memories (instances) in both grammaticality judgment and recognition tasks. However, they exhibited some difficulty in forming individual memories, as indicated by their greater tendency to mistakenly identify non-grammatical control items as previously seen during the training phase (recognition task). On untrained items, adults with dyslexia also showed an advantage with high compared to low-bigram frequency stimuli, indicating reliance on sublexical statistical cues to foster their performance. In conclusion, adults with dyslexia acquired procedural rules and showed sensitivity to the distributional properties of the stimuli, providing little support for the procedural hypothesis of dyslexia. This study highlighted both spared and compensatory mechanisms in adults with dyslexia. Previous conflicting results may depend on not having isolated which components (procedural vs declarative memory vs statistical learning) are involved in the AGL paradigm.

## Introduction

It has been proposed that a basic procedural deficit is the cause of various developmental disorders, including dyslexia [1–3]. The procedural deficit hypothesis posited by Ullman [1,2] draws heavily on the neurophysiological distinction between procedural and declarative processing. Procedural processing is mediated by brain structures including the basal ganglia, frontal cortex, parietal cortex, superior temporal gyrus, and cerebellum. In contrast, declarative processing is supported by the hippocampal region, the entorhinal and perirhinal cortex, and the para-hippocampal cortex. Procedural processing is essential for rule-based behavior, such as mental grammar, i.e., the complex of procedures underlying the combination of items into complex and hierarchical structures [1, 2]. Notably, procedures may be acquired through specific training but also implicitly by the learner, i.e., without the learner being aware of them. However, the precise distinguishing role of this factor is debated (e.g., [4]).

Numerous studies have employed various experimental paradigms that rely on procedural processing to test the hypothesis that individuals with dyslexia and other forms of developmental learning disorders [1–3] exhibit deficient procedural processing. For example, a widely used paradigm is the serial reaction time task [5,6]. Sequences of color or complex shapes are presented, including an unanticipated repeated sequence. Learning is marked by a reaction time (RT) advantage for the repeated sentence. Critically, cerebellar patients do not benefit from the repeated sequence, supporting the notion that the task assesses procedural processing [7]. An impressive number of studies using a variety of paradigms, including the serial reaction time (e.g., [5,6]), the Hebb repetition learning (e.g., [8]) and the weather prediction (e.g., [9] tasks), have tested this hypothesis. However, the body of evidence does not converge on a consistent pattern of findings. Thus, various recent reviews and meta-analyses propose contrasting conclusions on the hypothesis of a procedural deficit as a core deficit of dyslexia [10–14].

A distinction should be drawn between the ability to (implicitly) acquire a new rule and the ability to pick up probabilistic regularities in the environment (statistical learning). Statistical learning refers to the ability to detect regularities from quasi-regular systems and use this knowledge to predict future events (e.g., [15]). Many studies have related statistical learning abilities to different aspects of language acquisition: the ability to segment words from continuous speech [16], the acquisition of phonological and phonotactic categories [17], vocabulary acquisition [18,19], and more general language processing [20,21]. Several studies have also suggested a link between statistical learning and literacy skills in the typically developing population (e.g., [22–26]). Statistical learning skills facilitate reading and spelling [27]. Thus, letter sequences that have a high frequency of co-occurrence make it easier to read (e.g., [28–30]), write (e.g., [29–32]) and learn orthographic representations (e.g., [33]).

Both statistical learning and implicit skill learning have been framed within a procedural umbrella (e.g., [15]; including by Ullman et al. [2]). However, several indications suggest that learning probabilistic regularities and deterministic relationships may refer to distinct and dissociable mechanisms. For example, Quentin et al. [34] reported that high-order rules and statistical learning follow different consolidation paths, with the former occurring during rest periods while the latter occurs during practice. Furthermore, it has been proposed that the ability to pick up statistical properties may follow a different ontogenetic pattern than rule learning [35], showing a rapid decrement around the age of 12. Finally, the pattern of evoked potentials also distinguishes these two processes [36]. Overall, statistical learning has been framed as a broad construct that may refer not to a unitary mechanism but to a variety of different processes. These may be sensitive to various factors, such as the sensory modality of the stimuli, which rely on distinct neural structures [37]. Accordingly, statistical learning may impinge on procedural tasks but may also require declarative processes [15,37]. Although the placement of statistical learning relative to procedural learning may be a matter of debate, examining both probabilistic and deterministic mechanisms may be instrumental in framing the cognitive processes underlying learning disorders, particularly dyslexia.

A deficit in automatization is a critical feature of developmental learning disorders, particularly in regular orthographies, where children may be able to learn to read but do so in a slow, laborious fashion [38,39]. Critically, automatization has been framed in the literature from quite different theoretical perspectives. Within the procedural deficit hypothesis, automaticity is related to the rapid application of rules or procedures and, therefore, procedural memory [1–3].

However, there are alternative views of how automaticity is reached in over-practiced tasks. According to Logan’s “*instance theory of automaticity*” [40,41], individuals initially perform a task by applying an algorithm but, with practice, learn specific, i.e., item-based, solutions. Automatization would occur when, with sufficient practice, item-based retrieval responses become systematically faster than those based on the application of algorithms [40,41]. In this perspective, the automation difficulty in learning disorders might depend upon a deficit in declarative memory, i.e., a deficit in learning “*instances*” (or single events), and persistence in the use of algorithm-based procedures (such as sublexical reading, spelling, or counting procedures; see [42,43]). A relevant corollary of this interpretation is that the difficulty in learning instances may cut across learning disorders [44,45], contributing to their well-known comorbidity [46]. Consistently, a reduced capacity to form and consolidate individual “instances” is associated with low performance in tasks calling for item-based processing across different domains, such as performing an orthographic decision on pseudo-homophones, spelling irregular words, or retrieval of arithmetic facts [47]. We confirmed this pattern of results in the case of young adults with dyslexia [48]. Notably, this interpretation focuses on declarative, not procedural, memory.

Recently, we have proposed that jointly considering the predictions of dyslexia based on a procedural deficit [1–3] or on a reduced capacity to consolidate item-based memory [40,41] may allow a better understanding of the automatization deficit in learning disorders [49]. One task that may allow teasing out rule-based from item-based processing, as well as considering the influence of statistical learning, is Artificial grammar learning (AGL).

First introduced by Reber [50,51], the AGL task has often been used to examine the procedural hypothesis of dyslexia (e.g., [4,52–57]; see [13] for a meta-analysis). The AGL studies the ability with which participants implicitly learn stimuli containing regularities: sequences might be based on grammar that determines which sequences of stimuli can follow each other (grammatical stimuli, G) or not (non-grammatical stimuli, NG). We used the grammar rules developed by Ise et al. [53] to generate the stimuli of the present experiment. However, we replaced letters with non-orthographic materials to minimize the role of the orthographic inputs and merging processes (see below). AGL tasks [50,51] examine the ability of subjects to implicitly learn rules from repeated exposure to sequences of linguistic (e.g., letters [53–55]) or non-linguistic (e.g., abstract shapes [4,54,56,57]) visual stimuli. Participants must implicitly learn, by mere exposition, the statistical relationships within the letter strings and judge the grammaticality (i.e., rule compliance) of new items. According to the procedural deficit hypothesis [1–3], these processes may be like those involved in the implicit learning of literacy.

Several AGL studies reported learning deﬁcits in dyslexia among adults [56,58] and children [53,57,59]. However, many other studies did not show signiﬁcant eﬀects in children [54] or adults [4,55] with dyslexia. The presence of contradictory results may be due to differences in the type of tests used or variables considered in the experiment planning. Indeed, AGL tasks require the implicit acquisition of a rule set during the learning phase, but to be tested, implicit learning also requires novel items never seen before. However, some studies (e.g., [56]) in the testing phase (i.e., of grammaticality judgment) used target items learned during the training phase (which can also be solved based on instances and not necessarily on a procedural basis). So, it is not necessarily the case that the grammaticality judgment task is solved based on implicit rule learning. On the contrary, correct performance may also be achieved based on individual memory traces (instances). Therefore, these studies do not provide definite insights as to whether improvements depended upon processing by rule or instance acquisition (for a discussion, see [49]). Another confounding factor is statistical learning: often the relative contribution of rule learning and statistical learning have not been isolated, because grammatical test items typically have greater chunk strength than ungrammatical strings [60].

The present study aimed to evaluate whether performance in an AGL task depends on rule (procedural memory) or instance learning (declarative memory). Furthermore, we tested whether performance depended upon probabilistic regularities acquired during the learning phase (statistical learning) by including stimuli with a high vs a low bigram frequency of occurrence. This contrast would enable us to obtain information on the locus of deficit in the automatization process of adults with dyslexia. The study examined a group of young adults with dyslexia and a group of control participants with good literacy skills speaking Italian, a highly consistent orthography. Notably, there is no available evidence on AGL of individuals with dyslexia in this language.

In the training phase, studies generally resort to two possible methods: passive exposure [54,56,61] or active memorization (e.g., [53,55,62]). Active training can lead to better learning as participants focus more on the stimuli. Therefore, we opted for an active copying task during the training phase. After a learning phase, participants performed grammaticality judgment, recognition, and re-enactment tasks in writing. The first is an explicit task in which the learning of grammar rules is specifically, analytically and directly assessed. The second one is a more ecological task that focuses on retrieving the memory trace (i.e., instances) of learned items, but also indirectly assesses grammar rules. The third is a more complex re-enactment task that requires “*fully specified*” representations of the learned stimulus [63]. In every task, we used item manipulations to test whether participants performed the task through retrieval of instances from memory (items already learned in training vs new items), knowledge of implicitly learned grammatical rules (grammatical vs nongrammatical items), or acquisition of probabilistic properties (items with frequent vs atypical bigram frequency). We expected that this would provide insights into whether AGL tasks are performed primarily based on declarative or procedural memory and provide information on the locus of the deficit in developmental dyslexia.

Results from previous studies inform predictions about the performance of adults with dyslexia compared to control participants in the AGL paradigm for item-based, procedural and statistical learning processing, respectively. In learning studies, both children [47] and adults with dyslexia [48] demonstrated less improvement than controls in a task that required instance acquisition over repeated presentations. Additionally, their rate of learning in this task predicted how well they carried out arithmetic tables or retrieved lexical representations. For efficient performance, these tasks require efficient access to item-based information [47,48]. In contrast, there was no significant relationship between the rate of learning in the instance acquisition task and tasks that rely on algorithmic/procedural processing, such as judging numerosity or reading and spelling sub-lexically pseudowords [47,48]. Furthermore, various studies indicated a deficit in lexical expansion [64–66] and in acquiring lexical representations [67] in Italian children and adults with dyslexia. Based on all this evidence, we expected to find difficulties in instance acquisition and then poorer performance compared to controls with trained items across the different AGL tasks. More specifically, based on the hypothesis of lexical expansion deficit, we predicted that the deficit would be partial. Accordingly, we hypothesized that adults with dyslexia would show better performance when declarative memories could be used in addition to procedural memory and statistical distributional knowledge. Therefore, across tasks, we expected better performance with trained items (for which instances could be acquired, although not entirely efficiently) than with untrained items.

As stated above, the literature on a possible procedural deficit as a core deficit of dyslexia is vast, although punctuated by several failures to replicate. Accordingly, the various recent reviews and meta-analyses do not reach a consistent conclusion on this hypothesis [10–14]. In a previous paper [49], we proposed that for testing the procedural deficit hypothesis, it is relevant to consider both algorithmic/procedural and item-based processing within the same study. In previous studies making this type of control on Italian children, there was no indication that the slow rate of learning of children [47] and adults with dyslexia [48] was associated with procedural deficits. Based on this evidence, we anticipated that adults with dyslexia would show adequate acquisition of procedural information, as assessed by their ability to use acquired rules to solve the AGL tasks. Thus, we expected them to perform above chance not only on trained items (which can be solved based on either learning rules or instance memory) but also on untrained items (which require the acquisition of grammar rules). In the early phases of learning (as typical of AGL experiments), it proves easier to recognize grammatical items than reject non-grammatical ones [68]. Consistently, we expected better processing of untrained grammatical items compared to non-grammatical ones and that this tendency would also be detectable in adults with dyslexia, in keeping with their spared procedural processing.

Finally, previous studies revealed that both Italian children [29,30] and adults [65] with dyslexia used distributional knowledge. As a result, they were able to compensate in part for their lexical deficit [30]. Specifically, in both spelling-to-dictation and orthographic judgment, children with dyslexia were sensitive to the probability of sound–spelling mappings already by third grade [29]. When spelling words with unpredictable transcription (or making orthographic judgements on them), children with dyslexia did better with stimuli containing high-frequency (*i.e.*, typical) than low-frequency (i.e., atypical) sound-spelling mappings [30]. Notably, this tendency was more marked in children with dyslexia than in typically developing children. In a recent study using an orthographic judgement task under time pressure, adults with dyslexia processed irregular words with typical transcription more accurately, achieving an accuracy like that with regular stimuli, thus partially compensating for their lexical difficulties [65]. Therefore, based on this evidence, we expected adults with dyslexia to use probabilistic knowledge (i.e., as evidenced by better performance for high- than low-frequency bigrams), thus compensating for their difficulty in acquiring declarative memories (instances).

Note that these expectations were shaped within each of the four tasks used (learning task, grammaticality judgment task, recognition task and writing recall task), as in all tasks, we separately analyzed performance as a function of the type of stimuli. By contrast, we did not have explicit predictions on the overall performance in these various tasks. Quentin et al. [34] noted that overall performance in learning tasks (referred to as “general skill learning”) may depend on a variety of perceptual, cognitive, and motor skills, beyond procedural and declarative processing. Finally, for a comprehensive analysis of individual performance, we also considered other factors which are known to modulate performance. Thus, as targets comprised five-element strings, we examined the effect of position in the case of a rule violation. In fact, it is well-established that performance is lower for targets in intermediate positions compared to targets at the beginning and end of the symbol string [69]. This check allowed us to examine in an explorative way whether the key processes at play (procedural and declarative memories, or distributional knowledge) are particularly engaged when dealing with difficult items.

## Method

### Participants

Ninety-two undergraduate students from the University of Foggia participated in the study, including 32 adults with a diagnosis of dyslexia recruited from the learning disability office (mean age: 21.0 years) and 60 chronologically matched controls with typical literacy (mean age: 21.1 years). As can be seen from Table 1, the groups did not differ in gender, age, and scores on the Raven's Standard Progressive Matrices (SPM) [70].

**Table 1:** Socio-demographic variables of the sample.

	Adults with dyslexia		Controls			
	Mean	SD	Mean	SD	t test	p
Age (years)	21.13	3.56	21.02	3.27	t = 0.15	0.88
Raven SPMs (N correct responses)	42.84	6.69	43.17	14.41	t = 0.12	0.90
Gender	28 F, 4 M		52 F, 8 M		*X*^2^ = 0.01	0.91

Legend: SD = Standard deviation; F = Female, M = Male.

Informed written consent was collected from all subjects for participation in the study and processing of personal data to the goal of an open-access online publication. The subjects provided informed written consent to have data from their medical records used in research. The study was conducted following the principles of the Helsinki Declaration and approved by the Ethics Committee of Psychological Research of the University of Foggia (Decree No. 692 of July 30, 2021, of the Director of the Department of Humanities, University of Foggia, Prot. 011/CEpsi). Data collection of the study began on 05/05/2023 and ended on 09/30/2023, with the prior agreement of the Ethics Committee. The authors assert that all procedures contributing to this work comply with the ethical standards of the relevant national and institutional committees on human experimentation and with the Helsinki Declaration of 1975, as revised in 2008.

The diagnosis of a reading deficit was defined according to Delphi’s definition of dyslexia [71]. As reported by the panel, the manifestations of dyslexia may change over time, and some adults with dyslexia do not continue to experience word-level reading problems. However, they may have difficulties in reading, writing fluency and spelling. For this reason, in the group with dyslexia, we included adults with a deficit in reading accuracy or speed if present consistently while reading passages and single orthographic stimuli (words or pseudowords). Subjects with dyslexia had to meet the following criteria for inclusion in the experimental group: (a) performance of at least 1.65 standard deviations below the normative sample mean for speed and/or accuracy in reading a passage (LCS-SUA [72]); (b) performance of at least 1.65 standard deviations below the mean of the normative sample for speed and/or accuracy in reading at least one subset of the single word and pseudo-word reading test (LCS-SUA [72]); (c) performance within the norms at Raven’s Progressive Standard Matrices SPMs [70]. Spelling performance was not considered an inclusion criterion; however, as reported in Table 2, participants with dyslexia showed lower performance compared to control participants. Thus, 59.4% of dyslexic participants also showed a deficit in word and pseudo-word spelling (LCS-SUA [72]) and 37.5% in text spelling (LCS-SUA [72]).

**Table 2:** Results at the LCS-SUA battery [72] of the dyslexic and control groups. The data are z-scores, with negative scores indicating pathological performance.

	Adults with dyslexia		Controls			
	Mean	SD	Mean	SD	t test	p
Text comprehension	−1.55	1.11	−0.93	1.09	2.95	0.004
Text reading (errors)	−4.02	3.09	−0.74	1.08	7.54	0.000
Text reading (speed)	−1.14	1.14	−0.21	0.86	4.83	0.000
Word reading (errors)	−1.78	2.14	−0.00	0.72	5.97	0.000
Word reading (syll/sec)	−1.10	1.01	−0.06	0.89	5.61	0.000
Non-word reading (errors)	−1.80	1.90	−0.17	0.81	5.95	0.000
Non-word reading (syll/sec)	−0.97	0.81	−0.11	0.97	4.90	0.000
Lexical decision (ASC): correct score	−1.47	1.89	−0.24	1.08	4.28	0.000
Long HF word spelling (NC)	−0.97	3.22	0.18	0.71	2.76	0.006
Long LF word spelling (NC)	−1.41	2.17	−0.52	0.96	2.90	0.004
Long HF word spelling (ASC)	−0.99	2.02	0.26	0.58	4.61	0.000
Long LF word spelling (ASC)	−0.72	1.71	0.26	0.60	4.15	0.000
Text spelling	−1.00	1.66	0.04	0.89	4.22	0.000

Legend: NC = normal condition; ASC: articulatory suppression condition; HF = high frequency, LF = low frequency, SD = Standard Deviation.

A condition for inclusion in the control sample was the presence of performance in the normal range in (a) passage reading speed and/or accuracy (LCS-SUA [72]); (b) word and non-single word reading speed and/or accuracy (LCS-SUA [66]); (c) Raven’s SPMs [70]; and (d) absence of dyscalculia and dysgraphia (LCS-SUA [72]).

## Screening test

### Reading assessment

Reading ability was examined with the LCS-SUA test [72]. The following tests from the LCS-SUA battery [66] were individually administered to assess reading skills:

- *Text Passage Reading*: the subject is asked to read aloud a passage as fluently and correctly as possible. Two parameters are measured: (1) speed, the total time taken to read the passage is recorded and converted into syllables per second (sill. / sec.; 593/time in seconds); (2) accuracy, the total number of errors made. The following errors are penalized with 1 point: (a) inaccurate reading of the word (elision, substitution, insertion and/or inversion of syllable); (b) omission of syllable, word, or line; (c) addition of syllable, word, and re-reading of the same line; (d) pause longer than 5 seconds. On the other hand, the following errors are penalized with 0.5 points: (a) accent shifting; (b) self-correction for a 1-point major error (corrections for half-point errors are not considered); (c) major hesitation (i.e., a sounding-out behavior or initially inaccurate reading); (d) 1-point errors that do not change the meaning of the sentence. Repeated errors on the same word reappearing in the text are counted only once.
- *Word Reading*: The subject is asked to read aloud as quickly and accurately as possible four lists of words of different lengths and frequency of use. Each list consists of 4 subsets of 28 words each: (a) short (2- or 3-syllable) high-frequency words (SHF) each; (b) short (2- or 3-syllable) low-frequency words (SLF); (c) high-frequency long (4-syllable) words (LHF); (d) low-frequency long (4-syllable) words (LLF). Speed and accuracy are measured. The total time is calculated by summing the partial times obtained in the various subtests. The speed has been calculated by dividing the 352 syllables that make up the test by the total time taken to read the entire test (in seconds; 352 syllables/seconds taken). For accuracy, 1 point is awarded for each word omitted or incorrectly read. Incorrect use of stress is also considered an error. Autocorrections and strong hesitations are not considered errors.
- *Pseudoword Reading*: the subject is asked to read aloud two lists of pseudowords of different lengths. Each subtest consists of 28 pseudowords constructed by syllabic permutations of words in the word reading test. The subtests are divided into (a) short pseudowords composed of 2 or 3 syllables and (b) long pseudowords composed of 4 syllables. Speed and accuracy are measured. For the speed parameter, the syllables/second parameter is obtained by dividing the 176 syllables that make up the test by the total time (in seconds). For the accuracy parameter, 1 point is assigned to each pseudoword omitted or incorrectly read. Self-corrections and hesitations are not considered errors.

### Assessment of spelling

To assess spelling, the following tests from the LCS-SUA battery [72] were administered:

- *Word Dictation in standard and articulatory suppression conditions*: two lists of words were dictated at a constant pace. In the articulatory suppression condition, the student is asked to repeat the syllable “LA” out loud continuously during the task. In the standard administration, the examiner dictates eight lists of words in two different conditions: four in the standard condition (SC) and four in the articulatory suppression condition (ASC). In the present study, only long words (4/5 syllables) were dictated (for a total of four lists, two in the SC and two in the ASC). Each list consists of 14 words, varying in frequency.

In the standard condition, the examiner dictates words at a constant rate, typically about 3 seconds per word, but flexible to account for the spelling speed of each participant. In the articulatory suppression condition, the examinee is asked to continuously repeat the syllable “LA” aloud during the dictation task. Word dictation occurs at a pace as steady as possible, typically 3 seconds, but avoiding dictation if the student temporarily suspends articulation. If the student cannot follow the dictation rhythm, it is possible to pause and, subsequently, resume at a steady pace. One point is assigned to each misspelt, omitted, or incomplete word. Multiple errors on the same word are counted as only one error.

b. *Text Passage Dictation*: In this test, the examiner dictates a passage aloud, respecting established pauses and modulating the dictation rhythm according to the student's writing speed. It is possible to repeat a newly dictated term in special cases no more than two to three times. The total number of errors represents the score. One point is assigned to each misspelt, omitted, or incomplete word. We score only one error if a word is incorrect more than once.

Data from the screening tests are presented in Table 2. As can be seen from the table, the group of adults with dyslexia performed lower than controls in all domains of reading and spelling.

### Assessment of intellectual functioning

Analogical reasoning and abstraction skills were examined with the Raven SPM, following the standard procedure [70]. The Raven's SPM test is a non-verbal measure of fluid intelligence that uses a series of visual geometric patterns. Test-takers must identify the missing element in a matrix by analyzing the patterns and relationships within the other elements to determine which small image best completes it. The figure to be completed is always available to participants to minimize memory loading. The test assesses abstract reasoning, pattern recognition, and logical thinking without relying on language or cultural knowledge. The number of correct responses is the measure of performance. As shown in Table 1, the two groups of participants were comparable in this test.

### Experimental test

#### Stimuli.

The AGL task was developed from the matrix of grammar rules by Ise et al. [53]: all string sequences must start at “IN” and end at “OUT”. In our experiment, we replaced letters with non-orthographic symbols to minimize the role of the orthographic inputs (the conversion from letters to shapes is illustrated in Supporting Information).

**Fig 1:**
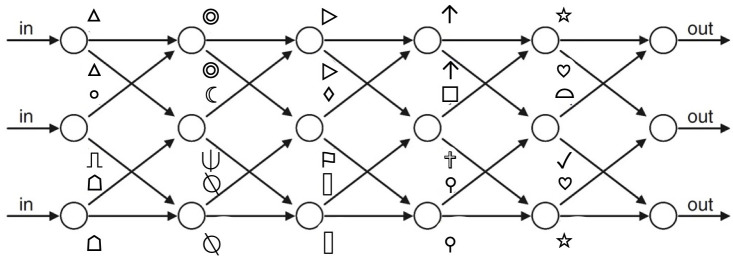
The artificial grammar employed in the present work. The grammar “rules” require that, given a particular prefix in a sequence of symbols, only certain continuations are allowed.

The grammar used is presented in Fig 1. The first symbols of the sequence must necessarily be △, ⚬, ⎍ or ☖. If a given sequence begins with the symbol △, the only possible transitions are the symbol ◎ or ☾. From the ◎ symbol, the transition may follow either with the symbol ▷ or ◊, depending on the directionality indicated by the arrow. From the symbol ☾, the transition may follow with the symbol ▷ or ⌷, and so on, until the end of the sequence (OUT), whose final symbols will necessarily be ☆, ♡, ⌓ or ✓.

Using the above pattern as a reference, all possible combinations (N = 95) of 5-symbol strings (e.g., △◎▷ ↑ ☆) were created, respecting the grammatical rules in Fig 1 that determine which stimuli may or may not follow each other (e.g., from ◎ the sequence may proceed to ▷ or ♢, but not return to △). We chose to use all strings of the same length (5 elements) so that length would not be a discriminating/facilitating factor.

Subsequently, we selected N = 20 stimuli from all possible strings for the learning phase. The frequencies of occurrence of each “bigram” were calculated from these stimuli. Then, we calculated the average frequencies of each of the remaining stimulus strings (the original 95 minus the 20 used in the learning phase) so that stimuli were distributed along a continuum from the most typical to the most atypical based on the distributional properties of the artificial grammar learned in the training phase.

After that, we selected the grammatical stimuli proposed in the grammatical judgment and recognition tests. Furthermore, we also generated non-grammatical stimuli (N = 60, half with low- and half with high-bigram frequency), which did not respect the succession rules (*i.e.*, the grammar arrows), introducing an error in the grammatical items by changing a single symbol in one of the five positions. Thus, 12 items had a grammar violation in the first letter, 12 in the second, and so on.

#### Procedure.

Stimuli were presented to participants in four different tasks (always performed in the same order by participants): (1) learning task (training phase), (2) grammaticality judgment, (3) recognition, and (4) writing recall.

To control for the effect of the stimuli repetition across tasks, two groups of students did not perform one task (in particular, 13 controls and 4 adults with dyslexia did not perform the grammatical judgment task, and 20 controls and 4 adults with dyslexia did not perform the recognition task). We compared their performance to that of participants who were given all three tasks with t-tests, but no significant differences emerged in any case (all ps > .05).

#### Learning task.

In the learning task (training phase), participants were exposed to a set of items that respected the rules of artificial grammar. Twenty symbol strings were used over eight learning trials. Strings were randomly presented one at a time in the center of a computer screen for an active copying task. Participants performed the copying task in the learning phase with all 20 stimulus strings. The stimuli randomization was different for each of the eight learning trials. Participants were exposed to stimuli and instructed to transcribe them. In particular, they were asked to carefully observe the string (composed of geometric figures) for 3 seconds (time required to ensure memorization) and copy it onto a sheet. A series of instructions was given to the participants. No other information on the grammatical nature, relation, form, or length of the stimuli was provided (a type of instruction typical of implicit learning paradigms). Then, for both correct and incorrect transcription, corrective feedback was presented with unlimited time. So, each participant was exposed to the stimuli 16 times, eight times with a limited time exposure during the copying phase and eight times with no time limit during the corrective feedback. If participants realized during corrective feedback that they had transcribed them identically, they were to mark “V”; if they had made mistakes or the string was incomplete, they were to mark “X.” The stimuli transcribed were covered after receiving the feedback so that the subjects could not carry out the test by looking at the sheet of stimuli already produced.

The scoring involved tallying the correctly copied items for each of the eight learning trials. In addition, a qualitative analysis of the type of errors produced was performed. Specifically, the errors were classified as follows: (a) reproduction of “*another string*” (the subject copied a different stimulus than the one presented); (b) “*correct but incomplete string*” (the subject copied the presented stimulus but incompletely, omitting some symbols from the string); (c) “*correct string but with 1 or 2 incorrect stimuli*” (the subject correctly copied the string excluding 1 or 2 incorrect stimuli); (d) “*string with some symbols in the wrong position*” (the subject correctly copied the string but reversed the position of some symbols); “I) “*no response*” (the subject did not copy any stimulus).

At the end of the training phase, participants were informed with a new set of “explicit” instructions that the stimuli seen previously were based on certain succession rules.

### Grammaticality judgment task

The assessment of learning grammar rules was conducted using a grammaticality judgment task, where participants had to indicate whether individual strings of symbols were grammatical or non-grammatical. This task aimed to evaluate participants’ ability to distinguish between strings that adhered to the rules of the learned artificial grammar (grammatical strings, G) and those that did not (non-grammatical strings, NG). To investigate whether participants based their responses on knowledge of grammar rules, distributional properties or learned instances, the following experimental conditions were established:

1. Trials subjected to training (N = 20): This subset included an equal split of strings with high and low bigram frequency. Participants could respond based on either grammar rules or specific instances learned during training.
2. New grammar trials with high bigram frequency (N = 20): This subset was expected to elicit responses based on on-rule learning, with performance potentially enhanced by distributional knowledge of the local frequency of co-occurring symbols.
3. New grammatical trials with low-bigram frequency (N = 20): Responses in this subset were anticipated to involve on-rule learning but with minimal assistance from distributional knowledge regarding symbol co-occurrence.
4. New non-grammatical trials with high-bigram frequency (N = 30): Errors in this subset might depend on the frequency of co-occurrence at the local level.
5. New non-grammatical trials with low-bigram frequency (N = 30): This served as a control condition.

To control for a possible response bias, half of the stimuli generated “YES” responses (subsets 1, 2 and 3) and half “NO” responses (subsets 4 and 5). Stimuli were randomized and presented in two blocks. Each block consisted of 60 stimuli (half generating “YES” responses and half “NO” responses), presented in three pages of two columns each.

Two squares were depicted next to each stimulus, one to be crossed out in case of a “YES” response, the other in case of a “NO” response. The subject’s task was to tick the “YES” box if the string complied with the rules of the artificial grammar learnt during training and “NO” if it did not. Scoring involved counting the accurate responses given by the participants for each experimental condition.

#### Recognition task.

In the recognition task, participants had to recognize which strings they had learned during training, and which were new stimuli never seen before. Again, there were different subsets:

1. Trials subjected to training (N = 80): This subset may be processed by referring to learnt instances during training. However, subjects may also erroneously use the acquired knowledge on grammar rules and use it to guide their judgement.
2. New grammar trials with high bigram frequency (N = 20). Errors in this subset might depend on learning the grammar (and erroneously taking a grammatical trial as one seen in the training phase) or by reference to the high frequency of local co-occurrence.
3. New grammatical trials with low-bigram frequency (N = 20): Errors in this subset might depend on using the grammar knowledge as a base for recognition.
4. New non-grammatical trials with high-bigram frequency (N = 20): Errors in this subset might likely depend upon reliance on the high-frequency co-occurrence at the local level.
5. New non-grammatical trials with low-bigram frequency (N = 20): Control condition.

To avoid the “yes” bias, we included an equal number of “YES” (subset 1) and “NO” (subsets 2, 3, 4 and 5) items. In subset 1, the learning stimuli were presented four times during the training phase to control for possible response biases and to assess the “verisimilitude” of learning. Indeed, by evaluating performance with the same stimuli, we can ensure that accuracy was not achieved by chance.

The stimuli were randomized and presented in two blocks. Each block consisted of 80 stimuli (half with a “YES” and with a “NO” response), presented in three pages of two columns each. Next to each stimulus, there were two boxes, one to be marked for a “YES” response and the other for a “NO” response. Participants were asked to tick the “YES” box if they recognized the string as one learned during the training phase and the “NO” box if the stimulus was new. Scoring was based on the accurate responses given by the participants for each experimental condition.

#### Writing recall task.

In the writing recall task, participants had to retrieve learned strings from memory and write down as many learned items as possible. The scoring involved counting the number of correctly re-enacted items and the number of errors for each typology (incorrect stimuli, omission of the entire string, order in the reported symbols in the string, and incomplete strings).

#### Data analysis.

We performed the analyses with the IBM SPSS 23 software [73]. The occurrence of implicit rule learning was assessed using one-sample t-tests comparing the performance of each group against chance level (50%) in the grammaticality judgment task and in the recognition task.

To compare the two groups of subjects, separate ANOVAs for the four tasks used were carried out on the percentage of accuracy with the group (adults with dyslexia vs controls) as a between-subjects factor. The within-subject variables considered in each ANOVA varied depending on the experimental test. The grammaticality factor was defined based on whether the stimuli involved (1) trained grammatical items (i.e., solvable by either learning the grammar rules or learning individual items or “*instances*”); (2) untrained grammatical items (i.e., solvable by learning the grammar rules); and (3) untrained non-grammatical items (control items). Furthermore, different analyses considered other factors, namely bigram frequency, number of item repetitions, and the position of violation within the string. Conditions are specified in the various analyses presented below. We explored significant interactions with Tukey’s *post-hoc* test. The types of errors in the learning phase and the spelling task were compared with ANOVAs with group as a between factor.

As indicated in the procedure, to control for the effect of stimulus repetition across tasks, some subjects did not carry out the grammaticality judgement and the recognition tasks. Furthermore, there were two missing data points in the writing recall task (two control participants). Accordingly, the overall N varied for the various ANOVAs, being N = 92 for the learning task, N = 75 for the grammatical judgement task, N = 68 for the recognition task and N = 90 for the spelling task.

## Results

### Learning task (training phase)

The ANOVA considered the group (adults with dyslexia, controls) as a between-subjects factor and learning trial (8 levels: from 1 to 8 learning trials) as a repeated measures factor. The learning trial’s main effect was significant (F _(7, 630)_ = 138.9; p < .0001; η^2^_p_ = .607): on the first learning trial, participants had low accuracy rates (26.8% accuracy), significantly lower than all other trials (at least p < .0001). Accuracy was around 86% from the 2^nd^ to the 5^th^ trial and then further increased to around 90% starting from the 6^th^ trial (at least p < .01).

The group main effect (F _(1,90)_ = 2.35; p = .13, η^2^_p_ = .025) and the group x learning interaction (F _(7, 630)_ = 0,90; p = .50, η^2^_p_ = .010) were not significant. Adults with dyslexia and controls had similar accuracy percentages in the learning test (77.9% and 83.3%, respectively). In the first trial, both groups encountered difficulties, while by the second trial, both controls and adults with dyslexia managed to respond correctly on most trials.

**Fig 2:**
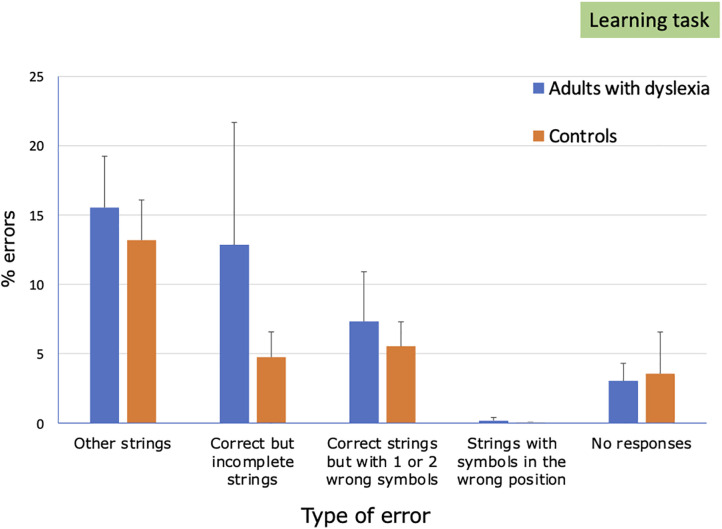
Different types of errors at the learning task (training phase). Data are separately presented for adults with dyslexia and controls. Errors indicate 95% confidence intervals.

The types of error produced by the two groups in the learning phase were compared with ANOVAs, with the group as a between-subjects factor and the different types of error as dependent variables (see Table 3 and Fig 2). The group effect was significant only in the case of the number of correct but incomplete strings (12.1% vs 4.4% for adults with dyslexia and controls, respectively). There were no significant differences between the two groups in all other types of errors. As can be seen from Fig 2, participants in both groups often reported a different string instead of the target one.

**Table 3:** ANOVAs on the different types of errors at the Artificial Grammar Test.

Type of error	F _(1,90)_	p	η^2^_p_
Other strings	1.65	.20	.018
Correct but incomplete strings	6.26	.01	.065
Correct strings but with 1 or 2 wrong symbols	1.01	.32	.011
Strings with symbols in the wrong position	3.27	.07	.035
No responses	.012	.91	.000

### Grammaticality judgment task

Results from one-sample t-tests showed that controls performed above chance level in the case of all three conditions (trained items: M = 86.60%, SD = 14.07 t _(46)_ = 17.83, p < .0001; untrained grammatical items: M = 85.32%, SD = 14.02, t _(46)_ = 17.24, p < .0001; and untrained non-grammatical items: M = 79.26%, SD = 14.02, t _(46)_ = 14.28; p < .0001). Furthermore, adults with dyslexia performed above chance level in all three conditions (trained items: M = 82.68%, SD = 15.42, t _(27)_ = 11.21, p < .0001); untrained grammatical items: M = 77.41%, SD = 13.43, t _27_ = 10.80, p < .0001; and untrained non-grammatical items: M = 69.58%, SD = 18.56, t _(27)_ = 5.58; p < .0001 = .001).

**Fig 3:**
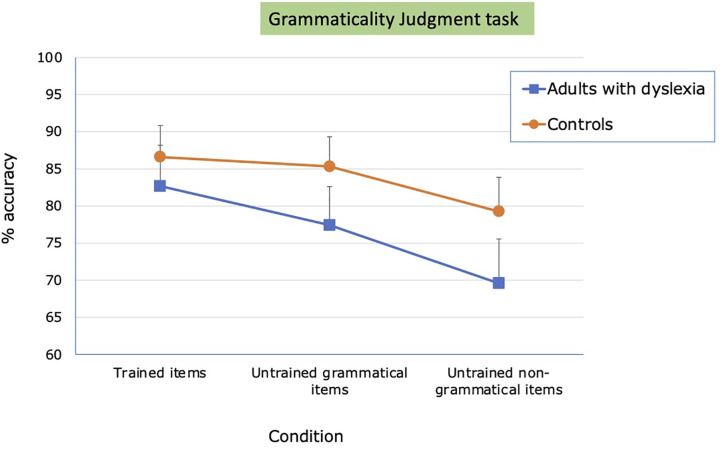
Effects of group and type of stimulus on the Grammatical Judgment task. The group x grammaticality interaction was not significant. Errors indicate 95% confidence intervals.

The ANOVA considered the group as a between-subjects group factor and grammaticality (learned items, non-learned grammatical items, non-learned non-grammatical items) as a repeated measures factor. The group effect was significant (F _(1,73)_ = 6.12; p < .05; η^2^_p_ = .077): adults with dyslexia were less accurate than controls in judging the string grammaticality (76.6% vs 83.7%, respectively). The grammaticality effect was also significant (F _(2,146)_ = 17.90; p < .0001; η^2^_p_ = .197): the trained stimuli were judged with greater accuracy (84.6%) than the new grammatical ones (81.4%, p < .01) and especially than the new non-grammatical ones (74.4%, p < .0001). The grammaticality effect did not interact with the group factor (F _(2,146)_ = 1.43; p = .24 η^2^_p_ = .019): although adults with dyslexia performed worse than controls, the two groups behaved similarly across conditions (see Fig 3). With trained stimuli, both groups had maximum accuracy; with new grammatical stimuli (not subjected to training), they had an intermediate accuracy; they performed worse with new non-grammatical stimuli.

#### Distributional properties.

On new items only, we examined the effect of distributional properties in interaction with item grammaticality. To this end, an ANOVA was conducted with the group as the between factor, and bigram frequency (high, low) and grammaticality (grammatical items, non-grammatical items) as repeated measure factors.

The ANOVA highlighted the significance of the group effect (F _(1,73)_ = 9.54; p < .01; η^2^_p_ = .116), with lower accuracy of adults with dyslexia compared to controls (73.5 vs 82.3%, respectively), and of the grammaticality effect (F _(1,73)_ = 10.69; p < .01; η^2^_p_ = .128), with lower accuracy in judging non-grammatical stimuli compared to grammatical ones (74.4% vs 81.4%, respectively).

**Fig 4:**
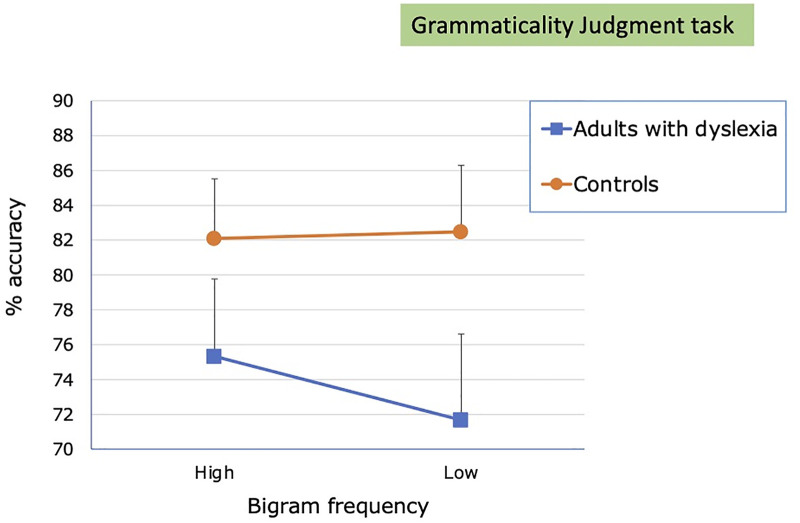
Effect of bigram frequency as a function of the group in the Grammatical judgment task. The group-by-bigram frequency interaction was significant. Errors indicate 95% confidence intervals.

The effect of bigram frequency tended towards significance (F _(1,73)_ = 3.50; p = .07; η^2^_p_ = .046) and interacted with the group (F _(1,73)_ = 5.37; p < .05; η^2^_p_ = .069). While in controls, performance was not modulated by bigram frequency (82.5 and 82.1% accuracy with low and high bigram frequency, respectively), in adults with dyslexia, performance was better for high bigram frequency (75.3%) than low bigram frequency items (71.7%; p < .01) (see Fig 4). This pattern did not interact with grammaticality, indicating that it was present for both grammatical and non-grammatical stimuli.

The group x grammaticality interaction was also not significant (F _(1,73)_ = 0.17; p = .68), indicating that both groups were more accurate with grammatical stimuli than with non-grammatical stimuli. No other interactions were significant.

#### Position of violation*.*

On non-grammatical items only, we analysed whether there were differences in how the two groups processed stimuli with grammatical violations depending on the violation position. The ANOVA considered the group as a between-subjects factor and bigram frequency (high, low) and violation position (1^st^, 2^nd^, 3^rd^, 4^th^, and 5^th^ position) as repeated measure factors.

The group effect (F _(1,73)_ = 6.52; p < .05; η^2^_p_ = .082) highlighted lower accuracy of adults with dyslexia (69.6%) compared to controls (79.3%). The violation position effect was also significant (F _(4,292)_ = 21.81; p < .0001; η^2^_p_ = .230), with lower accuracy in grammatical judgments with violations in the 3^rd^ and 4^th^ positions (68.2% and 67.4%, respectively) compared to the other positions (at least p < .0001) and greater performance with violations in 1^st^ position (81.4%; at least p < .0001). The violation position factor interacted with bigram frequency (F _(4,292)_ = 10.76; p < .0001; η^2^_p_ = .128): limited to stimuli with violations in the 3^rd^ and 4^th^ position, grammaticality judgements were better with stimuli with a high than low bigram frequency (at least p < .05); comparisons for all other positions were not significant, except to 2^nd^ position that showed the opposite pattern (p < .01).

All interactions with the group factor were not significant, indicating that the two groups performed similarly worse with the 3^rd^ and 4^th^ positions, especially for low-frequency bigram stimuli.

### Recognition task

Results from one-sample t-tests showed that controls performed above chance level in the case of trained items (M = 75.66%, SD = 18.36, t _(39)_ = 8.84, p < .0001) and untrained non-grammatical items (M = 70.69%, SD = 21.59, t _(39)_ = 6.06, p < .0001). In the case of untrained grammatical items, performance was significantly below chance level (M = 32.94%, SD = 18.14, t _(39)_ = −5.95; p < .0001). Subjects should have answered “NO” to these items, as these strings had never been seen before. However, they were presumably misled into answering “YES”, as items followed the same rules as the learned grammatical items. The pattern of results was similar for adults with dyslexia: they performed above chance level in trained items (M = 73.75%, SD = 13.98, t _(27)_ = 8.99, p < .0001) and untrained non-grammatical items (M = 62.05%, SD = 14.42, t _(27)_ = 4.42, p < .0001). In the case of untrained grammatical items, they performed significantly below chance level (M = 35.71%, SD = 16.09, t _(27)_ = −4.70; p < .0001).

The ANOVA considered the group as the between-group factor and grammaticality (learned items vs unlearned grammatical items vs unlearned non-grammatical items) as a repeated-measure factor. The grammaticality effect was significant (F _(2,132)_ = 104.72; p < .0001; η^2^_p_ = .613): accuracy was highest in recognising trained items (74.7%), lower in individuating untrained non-grammatical items (66.4%, p < .01); furthermore, it was much lower in identifying untrained grammatical items (34.3%, p < .0001). The group effect was not significant (F _(1,66)_ = 0.90; p = 0.35; η^2^_p_ = .013), as adults with dyslexia had an accuracy comparable to controls (57.2% vs 59.8%, respectively). The group x grammaticality interaction was not significant (F _(2,132)_ = 1.90; p = 0.15; η^2^_p_ = .028).

#### Stability of instance learning.

The trained stimuli were presented four times to the participants to check whether the performance was stable (as well as to control for a response bias). An ANOVA was conducted on only the learned items, with the group as a between-subjects factor and item repetition (1, 2, 3, and 4) as a repeated-measure factor.

The item repetition effect (F _(1,66)_ = 8.80; p < .01; η^2^_p_ = .118) highlighted higher accuracy for the 3^rd^ and 4^th^ time the same stimulus was presented to the participants (75.4% and 76.5%, respectively) than for the 1^st^ and 2^nd^ time seeing the stimulus (73.3% and 73.6% respectively, at least p < .05). The group effect was not significant (F _(1,66)_ =  .21; p = .65; η^2^_p_ = .003), as well as the item repetition x group interaction (F _(1,66)_ =  .92; p = .34; η^2^_p_ = .014): with repetitions, both groups increased in accuracy with increasing stimulus exposure.

#### Distributional properties.

The effect of distributional properties in interaction with item grammaticality could only be examined with new items. To this goal, we conducted an ANOVA with the group as between-subjects factor and bigram frequency (high vs low) and grammaticality (grammatical items vs non-grammatical items) as repeated measures.

The main effect of the grammaticality factor (F _(1,66)_ = 189.17; p < .0001; η^2^_p_ = .741) highlighted that the grammatical items were incorrectly recognised as previously learned, reporting lower levels of accuracy (34.3%) compared to the non-grammatical ones (66.4%). The group (F _(1,66)_ = 0.59; p = .45) and the bigram frequency main effects (F _(1,66)_ = 2.69; p = .11; η^2^_p_ = .039) were not significant, as the accuracy was comparable in adults with dyslexia and controls (48.9 vs 51.8%, respectively) and with high- and low-bigram frequency words (51.3% vs 49.4%, respectively).

**Fig 5:**
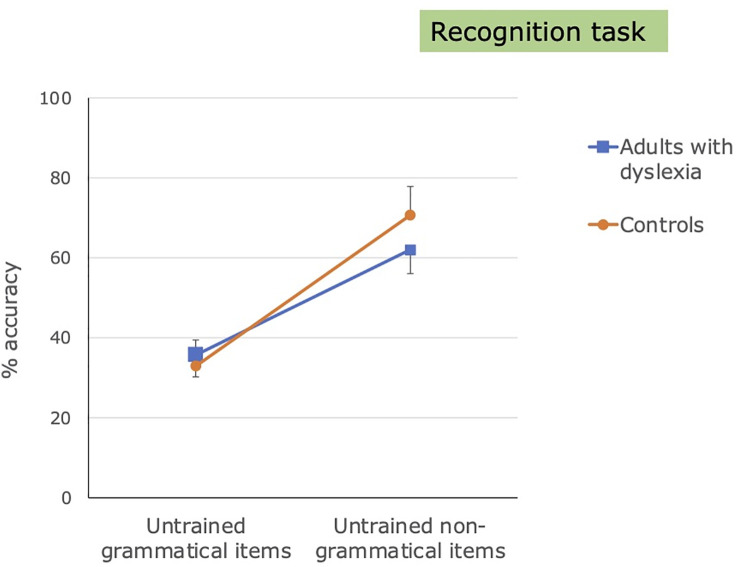
Untrained grammatical and non-grammatical items as a function of the group in the Recognition task. The grammaticality x group interaction was significant. Errors indicate 95% confidence intervals.

The grammaticality x group interaction was significant (F _(1,66)_ = 5.99; p < .01; η^2^_p_ = .083), indicating that, in the recognition of grammatical items, adults with dyslexia and controls had the same performance, while in the recognition of non-grammatical items, adults with dyslexia performed worse than controls (see Fig 5).

No other interactions were significant, including the bigram frequency x group interaction (F _(1,66)_ = 0.15; p = .70) and the grammaticality x bigram frequency x group interaction (F _(1,66)_ = 0.59; p = .44).

#### Position of the violation.

On non-grammatical stimuli alone, it was possible to evaluate whether there were differences in how the two groups processed stimuli with grammatical violations depending on the violation position. The ANOVA considered the group as between-subjects factor and bigram frequency (high, low) and violation position (1^st^, 2^nd^, 3^rd^, 4^th^, or 5^th^ position) as repeated measure factors.

The main effect of bigram frequency (F _(1,66)_ = 4.11; p < .05; η^2^_p_ = .059) indicated greater accuracy for stimuli with high- compared to those with low-frequency bigrams (68.0 vs 64.8%, respectively). The position of the violation factor was significant (F _(4,264)_ = 6.62; p < .0001; η^2^_p_ = .091), indicating differences in recognising stimuli with violations in 1^st^ (73.3%), 2^nd^ (67.9%) and last position (67.7%), compared with stimuli with violations in the 4^th^ position (63.6%, p < .05) and especially the 3^rd^ (where there was the worst performance with an accuracy of 59.3%; at least p < .01). The group effect tended towards significance (F _(1,66)_ = 3.41; p = .07; η^2^_p_ = .049), with lower accuracy of adults with dyslexia (62.1%) compared to controls (70.7%).

The interaction of bigram frequency x position of the violation was significant (F _(4,264)_ = 8.60; p < .0001; η^2^_p_ = .115): the effect of bigram frequency was stronger in the 4^th^ position compared to all other positions of the violation (p < .01). Thus, if the stimulus contained high-frequency bigrams, subjects were able to respond accurately in the case of a violation in a position in which they generally had greater difficulty.

The group did not interact with the violation position (F _(4,264)_ =  .48; p = .75; η^2^_p_ = .007) or with the frequency x position of the violation (F _(4,264)_ = 1.40; p = .24): both groups benefitted from the distributional properties and were advantaged in recognising stimuli with typical bigrams, especially if the violations were in the intermediate positions (where it was generally more difficult to identify them). No other interactions were significant.

### Writing recall task

The ANOVA with the group as the between-subjects factor did not highlight the significance of the group factor (F _(1,88)_ =  .041, p = .84; η^2^_p_ = .000): both adults with dyslexia and controls had low and similar performance in the writing recall task, managing to reproduce correctly, on average, 2.3 and 2.2 out of 20 stimuli, respectively. The error analysis highlighted no significant differences between groups for each error category (all Fs not significant): in most cases, dyslexic and control participants omitted the stimuli (mean = 10.5 and 8.9, respectively) or reported a stimulus different from the target (mean = 7.0 and 6.4, in each group, respectively). Incomplete stimuli were treasurable (mean of 0.1 for both groups), and errors in the order of the symbols were never present in either group.

## Discussion

We shaped our predictions within each of the four tasks (specifically as a function of the type of trials), and in the following, we will start discussing the pattern of results accordingly. For the sake of presentation, Appendix A presents a synthesis of the results, separately for the four tasks.

### Learning phase

In the learning phase, adults with dyslexia performed well, not showing a disadvantage compared to controls in the copying task and showing a similar increase in performance as the learning trials progressed. Accuracy increased for both groups as the number of presentations of the target stimulus increased, indicating greater confidence in the response provided as exposure and familiarity increased. The only difference between adults with dyslexia and controls was that the former produced more incomplete stimuli, omitting some symbols in the string. The challenges experienced may be due to difficulties in processing the stimulus within the three-second time limit, as well as issues related to working memory, the graphemic buffer, or visual analysis. This finding aligns with observations from previous studies (e.g., [74]). However, since participants consistently received corrective feedback, allowing them to view the entire string without any time restrictions, both groups should have had equal learning opportunities, which should not have influenced their performance on the subsequent tasks.

### Grammaticality judgment task

In the grammaticality judgment task, both adults with dyslexia and control participants performed above chance level, indicating that they had learned grammatical rules to some extent. The ANOVA on these data showed a significant grammaticality effect. Both adults with dyslexia and controls were more accurate in identifying grammatical stimuli as grammatical than in rejecting non-grammatical stimuli as non-grammatical (even though this latter judgement was also well above chance level). This finding is consistent with previous research on children with dyslexia, which shows that children tend to perform better with grammatical items than with non-grammatical ones (e.g., [57]; however, see [59] for different results). In the AGL paradigm, participants are presented with items from a specific category during the training phase and then asked to judge whether new exemplars belong to that category. It has been reported that the effect of grammaticality emerges most clearly when a sufficiently large set of grammatical exemplars is presented during the learning phase [68], as was done here. Apparently, learners form a relatively structured representation of what belongs to the target category. In contrast, their understanding of what does not belong to the category tends to be less defined [75], leading to lower performance on non-grammatical items.

Both groups of subjects were also more accurate with learned items than with grammatical ones. With learned items, the participant can carry out the task either based on declarative memory (acquired instances) and/or procedural knowledge (acquired grammatical rules). By contrast, with new grammatical items, the participant can solve the task only based on knowledge of the grammatical rules. The advantage for the learned items compared to the new grammatical ones indicates that both groups of subjects had at least in part acquired instances from the items seen in the training phase and benefited from this “lexical” knowledge. Therefore, both adults with dyslexia and controls took advantage of having two sources of convergent information for trained items (learned instances and grammatical rules) compared to a single source of information for novel grammatical and non-grammatical items (for which only procedural knowledge can support processing).

The gradient of difficulty based on the availability of information sources was consistent across groups, indicating that their processing was similar, as both benefited from having multiple information sources. However, adults with dyslexia were generally less efficient in completing this task. This group difference was unanticipated and, as such, may be open to different interpretations. We did not have explicit predictions concerning the overall performance, as this may depend upon a variety of perceptual, cognitive, and motor skills, beyond procedural and declarative processing, as stressed by Quentin et al. [34], who refer to this as “general skill learning”. In this vein, it is conceivable that general factors, such as short-term memory or attentional control (known to be associated with developmental dyslexia [76,77]), contributed to this finding. Possibly, the grammaticality judgment task may put a particular emphasis on control processes as the subject is required to formulate an explicit dichotomous decision on each symbol string. Thus, in interpreting the poor performance of adults with dyslexia in the grammatical judgment task, one possibility is that the difficulty is associated with a deficit in explicit awareness of grammaticality, not in tasks requiring implicit knowledge. Thus, the lack of explicit awareness of grammaticality could be linked with a general metacognitive deficit in awareness, often found in participants with dyslexia [78,79]. Further research is warranted to test this possibility.

Alternatively, it is possible to posit that the group difference was indeed associated with the factors intrinsic to the performance on the grammar task. In this vein, consider that the inefficiency of adults with dyslexia spanned across items requiring item-based and rule-based processing. So, to use this piece of information as proof of weak procedural learning, one should also posit that adults with dyslexia are homogeneously impaired in both item-based and rule-based processing, as the pattern of the grammaticality effect was very similar in the two groups. As there is consolidated evidence that these two types of processing refer to distinct and independent mechanisms [1,2,40,41], we find this interpretation unlikely, although it cannot be dismissed with certainty.

In the grammaticality judgment task, there was also evidence of the influence of distributional properties (as indicated by bigram frequency, i.e., the relative frequency of the sequence of two symbols presented). On new (grammatical and non-grammatical) items, adults with dyslexia showed an advantage with high compared to low bigram frequency stimuli, indicating that they could rely on sublexical statistical cues to foster their performance. This advantage was not evident among controls. However, the lack of a bigram frequency effect in typically developing readers does not necessarily imply that they did not use bigram frequency. By contrast, it might relate to their generally higher performance, making the advantage for high-frequency bigram stimuli less needed. For non-grammatical items, we also investigated how distributional properties interacted with the position of violation. The data confirmed the established finding that performance tends to be lower in intermediate positions, other than the foveal one [69], a pattern observed in both groups of subjects. For these more challenging items, both adults with dyslexia and control participants exhibited better performance with high bigram frequency stimuli than with low frequency ones, demonstrating their sensitivity to this distributional property. Overall, both groups of subjects were capable of using probabilistic knowledge to enhance their performance efficiency when needed.

### Recognition task

In the recognition task, subjects had to identify the targets seen during the training phase, regardless of whether they were consistent or not, with the grammar rules. However, the data clearly indicated that knowledge of grammar rules intruded on the recognition judgement. Thus, for both the dyslexic and control groups, the presence of new grammatical items proved misleading in recognizing stimuli previously subjected to training. In these items, participants should have answered “NO” as they never saw these strings before. However, presumably, they were misled into answering “YES”, as items followed the same rules as the learned grammatical items. Thus, with grammatical items, both groups performed worse because they mistook them for correct. Notably, this confounding effect with grammatical stimuli was present in both groups, indirectly suggesting that adults with dyslexia had also learned the grammar rules, like controls. Note that an increased bias toward rule-based reasoning may also depend upon having taken the recognition test after the grammaticality judgment task and from having made explicit the existence of grammatical rules after the learning task (e.g., 58; but see 57 for contrasting results). Accordingly, a note of caution is in order.

The results of the recognition task indicate that both groups of participants acquired the items presented during the learning phase and recognized them to some extent. Neither group solved the task by chance, as highlighted also by the analysis examining performance stability for learned trials. They showed a general increase in accuracy with the repetition of learned items, presumably due to an increasing familiarity with the stimuli and confidence in the response as exposure to them increased. However, this item repetition effect did not interact with the group effect, showing a similar pattern as a function of item repetition between adults with dyslexia and control participants. Therefore, adults with dyslexia, as controls, have also learned, to some extent, individual items, forming instances. Adults with dyslexia, however, made more errors in rejecting non-grammatical control items. Thus, they mistakenly took them as previously seen during the training phase more often than control participants (for whom these stimuli proved not so confounding). Conceivably, adults with dyslexia may have developed less stable instance representations than control participants, with the consequence of being more easily led into doubt about the new stimuli. However, this interaction was significant only in the sub-analysis on new items, which examined the impact of distributional properties, but failed to reach significance in the general analysis considering all stimuli.

Also in the recognition task, both groups were influenced similarly by the probabilistic properties and more easily recognized strings with more frequent bigrams. This finding indicates a spared capacity to learn the distributional properties of the stimuli to which they were exposed. They also used this knowledge to compensate for dealing with challenging targets. Thus, recognizing stimuli with grammatical violations was worse in intermediate (3–4) positions, consistent with data on letter perceptibility in visual recognition (e.g., [80]). However, both adults with dyslexia and controls compensated for this difficulty by using their knowledge of distributional properties, thereby improving their performance with high-frequency bigram stimuli.

### Writing recall task

In the writing recall task, adults with dyslexia and controls performed similarly and, in general, quite poorly. It is, therefore, considerably more difficult to recall a stimulus in writing than to recognize it since, as already indicated, “*fully specified”* representations are necessary in writing (more than in reading) [63]. Thus, the participants learned the grammatical rules and could recognize stimuli subjected to training by recalling the instances, but did not have representations detailed enough to allow adequate recall in writing. Note that no significant difference emerged between groups in the type of errors produced in spelling. In most cases, stimuli were either omitted or errors consisted of stimuli different from those learned, while the number of incomplete strings was very low. Errors in the order of symbols in the string were not present.

### General remarks

Overall, the results showed several pieces of evidence that adults with dyslexia learned the artificial grammar to some extent and relied on procedural memories much like control participants. During the learning phase, adults with dyslexia showed improvement in their performance as the trials progressed, much like the control group. In the grammaticality judgment test, they demonstrated above-chance performance in judging the grammaticality of new (previously unseen) items. Additionally, they exhibited a grammaticality effect in the grammaticality judgment task, favoring grammatical items over non-grammatical ones, as did controls. At least during the initial phases of acquiring a new grammar, it is known that learners develop a more structured representation of which items belong to the target category compared to those that do not [75]. In the recognition task, adults with dyslexia were confused by grammatical items, mistakenly identifying them as previously encountered during the learning phase. This confounding effect of grammatical stimuli was similar to that observed in control participants.

We also observed that adults with dyslexia acquired and resorted to distributional properties. In fact, like controls, they compensated for the difficulties when the rule violation was in a critical position (3–4) in the case of stimuli with high bigram frequency in the grammaticality judgment and recognition tasks. Additionally, adults with dyslexia reduced errors in grammaticality judgment with high-frequency stimuli to a greater extent than controls. Thus, adults with dyslexia made marked use of distributional properties to compensate for their difficulties, consistent with previous studies on children [29,32,47,54,81] and adults with dyslexia [82]. Statistical learning has been framed as part of a large family of procedural processing [2,15,60], and several studies have tested the procedural deficit hypothesis [1,2] based on statistical learning (e.g., [54]). To the extent that statistical learning is informative of procedural processing, the present results indicate that adults with dyslexia show adequate sensitivity to distributional properties and, indeed, rely on this piece of information more than control participants, particularly in the case of difficult items. Therefore, this pattern of findings is also inconsistent with the procedural deficit hypothesis, at least in its original formulation [2].

However, there were also indications that adults with dyslexia might be less efficient in acquiring or utilizing procedural information compared to their peer controls. The seemingly most compelling piece of evidence for this comes from the observation that adults with dyslexia performed more poorly in the grammaticality judgement task. In contrast, this was not the case in the other three tasks used in the study. This inefficiency affected items requiring both item-based and rule-based processing. Therefore, to interpret this as evidence of weak procedural learning, we would need to assume that adults with dyslexia are impaired uniformly in both types of processing. However, there is consolidated evidence that these two types of processing rely on distinct and independent mechanisms [1,2,40,41]. Therefore, this interpretation seems unlikely, although it cannot be entirely dismissed. Alternatively, we propose that, in complex tasks, a range of perceptual, cognitive, and motor skills – beyond procedural and declarative processing – may influence overall performance (for a discussion on learning tasks see [34]). Consequently, overall group differences may be related to factors such as short-term memory or attentional control, which are known to be associated with developmental dyslexia [76,77]. In the recognition task, the participant may carry out the task solely based on stimulus familiarity; in contrast, performing the grammaticality judgment task may require more controlled and analytical processes (and therefore, mnemonic and attentional processes are more involved). Above, we have proposed that the poor performance of adults with dyslexia in the grammatical judgment task, but not in recognition and writing tasks, may be associated with a general metacognitive deficit in awareness, often found in participants with dyslexia [78,79]. Further research is warranted to test this possibility.

Overall, the present pattern of findings provides, at best, very weak evidence for the idea that developmental dyslexia stems from a domain-general deficit in acquiring procedural memories, as proposed by Ulmann [1,2] and Nicolson and Fawcett [3]. This observation does not exclude the possibility that individuals with dyslexia may struggle with the specific procedural aspects associated with learning to read. We know that acquiring grapheme-to-phoneme is a key requisite of reading acquisition. In the absence of strong evidence supporting the procedural hypothesis, it is reasonable to adopt a cautious approach and assume that the procedural challenges faced by individuals with dyslexia are specific to this task rather than indicative of a broader procedural deficit.

Furthermore, adults with dyslexia acquired and utilized memories of individual learned strings (instances), as indicated by better performance with these learned items in both grammaticality judgment and recognition tasks. However, in the recognition task, adults with dyslexia experienced more marked difficulties than control participants in rejecting non-grammatical stimuli that were often confused with trained items. Therefore, there is an indication that adults with dyslexia have developed less stable instance representations, making it difficult for them to judge new stimuli in the recognition task. This finding appears consistent with studies indicating poor lexical processing and acquisition among adults with dyslexia [83,84]. A meta-analysis of adults with dyslexia [85] revealed that particularly high-functioning individuals could use phonological skills, but their main difficulty was memorizing orthographic patterns [86]. Note that while the AGL effectively identifies the acquisition of instances, it has an inherent limitation: acquisition occurs over a much shorter time frame than what typically happens during literacy acquisition. This limitation is particularly evident in the low performance in the writing recall task. Consequently, the current findings relate only to the initial stages of instance acquisition. Understanding long-term learning would require further studies designed over an extended time frame. Additionally, note that results pertain specifically to a high-consistency orthography (Italian). Previous evidence indicates that Italian readers activate the lexical procedure, although to a lesser extent than speakers of an irregular orthography, such as English [87–92]. It would be interesting to assess the performance of English speakers in an AGL task, such as the one developed in the present study.

In conclusion, artificial grammar tasks may be performed based on different types of information: procedural (knowledge of the rules), probabilistic (sensitivity to distributional properties), and declarative (learned instances). As such, this paradigm may potentially represent a microcosm of the processes leading to reading acquisition. The data from the present research highlighted that all these processes were involved. Thus, it was informative to examine all these processes separately for acquiring information on the possible locus of deficit in students with dyslexia [49]. Data indicated that adults with dyslexia acquired procedural rules and showed sensitivity to the distributional properties of the stimuli. They used these sources of information to compensate for their difficulties and enhance their performance, although they still exhibited deficiencies in certain conditions. This pattern of findings does not align well with the hypothesis that dyslexia is due to a domain-general difficulty in acquiring rule-based processing, as stated by the procedural deficit hypothesis [1–3]. Additionally, adults with dyslexia benefited from using declarative memory to solve tasks, which helped them improve their performance in grammaticality judgment and recognition tasks involving learned items, even with new, untrained items. However, they also showed some limited difficulty forming and consolidating individual memories (instances), as indicated by the greater tendency to mistake control items with non-grammatical errors for learned items. This finding provides partial support to the idea that the automation deficit exhibited by these individuals may be associated with a deficient ability to consolidate instances [43,44,47]. It highlights the need for adults with dyslexia to have greater exposure to acquiring orthographic representations compared to typical readers [66]. We propose that the discrepant results in the literature on the procedural deficit hypothesis might at least partly depend on the fact that no study has ever jointly examined all these processes involved in carrying out AGL tests. It would be interesting to replicate this study (considering simultaneously all processes involved) on children with developmental dyslexia, for whom fewer compensatory mechanisms may likely be in action.

## Supporting information

S1 Appendix Appendix A. The table synthesizes the main findings of the study in relationship to the different tasks. Size effects (η^2^_p_) for significant main effects and interactions are reported. Stimuli used for the Artificial grammar learning task.
(DOCX)

S1 File Supporting information. (DOCX)
