# Supplementary material for: Procedural or declarative deficit in adults with developmental dyslexia? A study of Artificial grammar learning
Source: PLoS One. 2026 Jul 29;21(7):e0352337. doi: 10.1371/journal.pone.0352337 (PMC13419226; doi:10.1371/journal.pone.0352337)
Supplement: S1 Appendix — The table synthesizes the main findings of the study in relationship to the different tasks. Size effects (η2p) for significant main effects and interactions are reported. Stimuli used for the Artificial grammar learning task. (DOCX) [file pone.0352337.s001.docx]

**Appendix A**

The table synthesizes the main findings of the study in relationship to the different tasks. Size effects (η^2^_p_) for significant main effects and interactions are reported.

| **Task** | **Effects/interactions** | **Size effect η^2^_p_** | **Main findings (errors)** | **Description** | **Core deficit vs spared skills in adults with dyslexia** |
| --- | --- | --- | --- | --- | --- |
| **Learning task** | Group | - | Dysl. = controls |  | Spared learning skills |
|  | Learning trial | .607  (large) | Accuracy improves with trials |  |  |
|  | Learning trial x Group | - | Groups improve similarly with trials |  |  |
| **Grammaticality Judgment task** | Above chance |  | Dysl. and controls for all stimuli |  |  |
|  | Group | 0.77  (medium) | Dysl. > controls | Dysl. underperformed in this task with respect to controls, but the group effect did not interact with stimulus type: their difficulty was generalized and spanned across items requiring both item-based and rule-based processing. | Deficit in making grammatical judgments but generalized and not restricted to grammatical stimuli. |
|  | Grammaticality | .197  (large) | New ungrammatical > new grammatical > trained | Both groups performed better with trained items (that are supported by the learning of instances and grammatical rules) compared to untrained ones. | Multiple sources of information support processing. |
|  | Grammaticality x group | - | New ungrammatical > new grammatical for both groups | Both groups performed better with novel grammatical items (for which procedural knowledge can support processing) compared to non-grammatical ones. | Spared acquisition of grammatical rules. |
|  | Bigram frequency | .046*  (small) |  |  |  |
|  | Bigram frequency x group | .069  (medium) | Low > high bigram frequency only in dysl. | Only dysl. were advantaged with high-bigram frequency stimuli | Spared statistical learning |
|  | Bigram frequency x group x grammaticality | - | Low > high bigram frequency only in dysl. irrespective of grammaticality |  |  |
|  | ***Position of violation (on non-grammatical stimuli only)*** | | | |  |
|  | Group | .082  (medium) | Dysl. > controls |  |  |
|  | Position | .230  (large) | 3°-4° > 1°-2°-5° |  |  |
|  | Position x bigram frequency | .128 (medium) | Difficulty for 3°-4° positions compensate for high-bigram stimuli |  |  |
|  | Position x group | - | Groups performed similarly worse with the 3rd and 4th positions, especially for low-frequency bigram stimuli. | Dysl. were advantaged with high-bigram frequency stimuli, with new stimuli; both groups compensated for the difficulty in intermediate positions for high-bigram frequency stimuli. | Spared statistical learning and use of these competencies to compensate for difficulties. |
|  | Bigram frequency x group | - |  |  |  |
|  | Position x bigram frequency x group | - |  |  |  |
| **Recognition task** | Above chance |  | Dysl. and controls for trained and untrained ungrammatical stimuli |  |  |
|  | Below chance |  | Dysl. and controls for untrained grammatical stimuli |  |  |
|  | Grammaticality | .613  (large) | grammatical items > non-grammatical > trained items | For both groups, knowledge of grammar rules intruded on the recognition judgement of grammatical stimuli. | Spared acquisition of grammatical rules |
|  | Group | - | Dysl. = controls |  |  |
|  | Group x grammaticality | - | Groups behaved similarly across conditions |  |  |
|  | ***Stability of instance learning (on trained items only)*** | | | |  |
|  | Item repetition | .118 (medium) | 1°-2° times > 3° 4° times | Both groups acquired trained trials (and increased their confidence in the response with item repetitions) | Spared instances acquisition, but less stable instance representations. |
|  | Group | - | Dysl. = controls |  |  |
|  | Group x | - | Groups behaved similarly across conditions |  |  |
|  | ***Distributional properties (on new items only)*** | | | |  |
|  | Group | - | Dysl. = controls |  |  |
|  | Grammaticality | .741  (large) | grammatical > ungrammatical |  |  |
|  | Bigram frequency | - | high = low bigram frequency |  |  |
|  | Grammaticality x group | .083  (medium) | for grammatical: Dysl. = controls; for ungrammatical: Dysl. > controls | However, dysl. had difficulty in rejecting non-grammatical items | Spared statistical learning and use of distributional propriety as a compensatory strategy for more difficult stimuli. |
|  | Bigram frequency x group | - |  |  |  |
|  | Bigram frequency x group x grammaticality | - |  |  |  |
|  | ***Position of violation (on non-grammatical stimuli only)*** | | | |  |
|  | Group | .049*  (small) | Dysl. > controls | Both groups compensated for their difficulty in recognizing stimuli with grammatical violations in intermediate positions by using knowledge of distributional properties and improving their performance with high-frequency bigram stimuli. | Spared statistical learning and use of distributional propriety as a compensatory strategy for more difficult stimuli. |
|  | Bigram frequency | .059  (small) | low > high bigram frequency |  |  |
|  | Position | .091  (medium) | 3°-4° > 1°-2°-5° |  |  |
|  | Position x bigram frequency | .115  (medium) | Difficulty for 4° position compensated for high-bigram stimuli |  |  |
|  | Position x group | - | Groups performed similarly worse with the 3rd and 4th positions, especially for low-frequency bigram stimuli. |  |  |
|  | Bigram frequency x group | - |  |  |  |
|  | Position x bigram frequency x group | - |  |  |  |
| **Writing recall task** | Group | - | Dysl. = controls |  | Similar impaired recall in both groups. |

Legend: Dysl. = Adults with dyslexia; * Approached to the significance; Column main finding refers to errors data for each condition and group.
