# Supplementary material for: Procedural or declarative deficit in adults with developmental dyslexia? A study of Artificial grammar learning
Source: PLoS One. 2026 Jul 29;21(7):e0352337. doi: 10.1371/journal.pone.0352337 (PMC13419226; doi:10.1371/journal.pone.0352337)
Supplement: S1 File — (DOCX) [file pone.0352337.s002.docx]

**Supporting Information**

**Artificial grammar stimuli**

| FXDWZ | △⍦⌷⚲☆ |
| --- | --- |
| JXDHZ | ☖⍦⌷✞☆ |
| MPDWN | ⎍⍉⌷⚲♡ |
| JSGHN | ☖☾▷✞♡ |
| FTVCQ | △◎♢↑⌓ |
| FTGCN | △◎▷↑♡ |
| JPKWN | ☖⍉⚐⚲♡ |
| JPVCB | ☖⍉♢↑✓ |
| FXDWB | △⍦⌷⚲✓ |
| FSGLN | △☾▷☐♡ |
| FSGHN | △☾▷✞♡ |
| MPVCB | ⎍⍉♢↑✓ |
| JPDWZ | ☖⍉⌷⚲☆ |
| JXDWN | ☖⍦⌷⚲♡ |
| RTGCB | ⚬◎▷↑✓ |
| RTGLN | ⚬◎▷☐♡ |
| MPVCN | ⎍⍉♢↑♡ |
| MPKWQ | ⎍⍉⚐⚲⌓ |
| FTKWN | △◎⚐⚲♡ |
| JSGCZ | ☖☾▷↑☆ |
